# Discriminative Identification of SARS-CoV-2 Variants Based on Mass-Spectrometry Analysis

**DOI:** 10.3390/biomedicines11092373

**Published:** 2023-08-24

**Authors:** Liron Feldberg, Anat Zvi, Yfat Yahalom-Ronen, Ofir Schuster

**Affiliations:** 1 Department of Analytical Chemistry, Israel Institute for Biological Research (IIBR), Ness Ziona 74100, Israel; 2 Department of Biochemistry and Molecular Genetics, Israel Institute for Biological Research (IIBR), Ness Ziona 74100, Israel; anatz@iibr.gov.il; 3 Department of Infectious Diseases, Israel Institute for Biological Research (IIBR), Ness Ziona 74100, Israel; yfatyr@iibr.gov.il

**Keywords:** COVID19, SARS-CoV-2, variants of concern, diagnosis, mass-spectrometry, LC-MS/MS, spike, nucleocapsid

## Abstract

The spread of SARS-CoV-2 variants of concern (VOCs) is of great importance since genetic changes may increase transmissibility, disease severity and reduce vaccine effectiveness. Moreover, these changes may lead to failure of diagnostic measures. Therefore, variant-specific diagnostic methods are essential. To date, genetic sequencing is the gold-standard method to discriminate between variants. However, it is time-consuming (taking several days) and expensive. Therefore, the development of rapid diagnostic methods for SARS-CoV-2 in accordance with its genetic modification is of great importance. In this study we introduce a Mass Spectrometry (MS)-based methodology for the diagnosis of SARS-CoV-2 in propagated in cell-culture. This methodology enables the universal identification of SARS-CoV-2, as well as variant-specific discrimination. The universal identification of SARS-CoV-2 is based on conserved markers shared by all variants, while the identification of specific variants relies on variant-specific markers. Determining a specific set of peptides for a given variant consists of a multistep procedure, starting with an in-silico search for variant-specific tryptic peptides, followed by a tryptic digest of a cell-cultured SARS-CoV-2 variant, and identification of these markers by HR-LC-MS/MS analysis. As a proof of concept, this approach was demonstrated for four representative VOCs compared to the wild-type Wuhan reference strain. For each variant, at least two unique markers, derived mainly from the spike (S) and nucleocapsid (N) viral proteins, were identified. This methodology is specific, rapid, easy to perform and inexpensive. Therefore, it can be applied as a diagnostic tool for pathogenic variants.

## 1. Introduction

Since the emergence of SARS-CoV-2 in late 2019, new variants of concern (VOCs) have emerged, leading to a prolonged battle against COVID-19. Among them are the variants Alpha, Beta, Gamma and Delta, originating from the UK, South Africa, Brazil and India, respectively. The spread of a new VOC has significant implications for public health, since genetic changes may increase virus transmissibility and disease severity. Furthermore, it may reduce the effectiveness of vaccines or lead to diagnostic failures [1,2]. Single nucleotide polymorphisms in the SARS-CoV-2 RNA may cause false negative results in the diagnosis of the virus using genetic methods, originally developed for the wild-type strain (Wuhan reference strain) [3]. RT-PCR, the gold-standard method for SARS-CoV-2 detection, may yield false negative results when the mutations interfere with primer binding [4,5]. A technical document summarizing the possible methods for identifying variants was recently published by the World Health Organization [6]. Whole Genome Sequencing (WGS), or at least complete or partial spike (S)-gene sequencing, is the best method for characterizing a specific variant. Diagnostic screening nucleic acid amplification technique (NAAT)-based assays are used as an alternative method that enables the early detection and pre-screening of VOCs. Recently, PCR-based studies have been published to address the diagnosis of SARS-CoV-2 variants [7,8,9,10,11]. Holand et al. showed that the use of digital PCR will enable the need for a personalized diagnosis aimed at individual patient treatment [8], and Vogels et al. aimed to reduce false negative results by multiplexed RT-PCR, developing a set of dedicated primers for variants sharing common mutations [12].

Mass spectrometry (MS) methods for SARS-CoV-2 identification, based on unique peptide markers, have been reported since the beginning of the pandemic [13,14,15,16,17]. In our previous studies, we demonstrated a rapid SARS-CoV-2 identification assay using mass spectrometry (LC-MS/MS). The assay is based on the identification of six SARS-CoV-2-specific peptides, four of which are derived from the spike protein (SFIEDLLFNK, FLPFQQFGR, FQILLALHR and HTPINLVR), and the other two are derived from the nucleocapsid protein (GFYAEGSR and AYNVTQAFGR) [16]. An improvement of the assay’s sensitivity up to 1 × 10^3^ PFU/mL was achieved by adding a concentration and purification step using immunomagnetic beads coated with anti-SARS-CoV-2 antibodies, combined with a sensitive MRM-based LC-MS/MS analytical method for the target markers [17].

With the emergence of VOCs and the accumulating knowledge base regarding mutations occurring during the spread of the virus, sequence conservation of these peptide markers among the different variants became a fundamental requirement for their continued use in SARS-CoV-2 diagnostic assays. Therefore, inter-strain conservation was one of the main criteria for marker selection in our previous study [16]. However, in addition to the prevention of false negative results in diagnostics, the different infectivity and breakthrough infection rates for these new VOCs make the identification of the specific variant of great importance. To date, genetic sequencing is the gold-standard method to discriminate between variants. Yet, it is time consuming (taking days) and relatively expensive [18,19]. Recently, researchers have begun to evaluate the efficacy of MS-based proteomics approaches for the detection of SARS-CoV-2 variants of concern. Mann et al. pioneered MALDI-FT-ICR MS, a peptide mass-fingerprinting method, to detect the Alpha, Beta, Gamma and Delta variants. As a peptide mass-fingerprinting approach, MS/MS spectra were not acquired, and the method required the isolation of the S-protein. They successfully measured the masses of mutated peptides and constructed a phylogenetic tree to track variant evolution [3]. Maus et al. highlighted the importance of including variants of concern in assay design and detected variant peptides from the N and ORF1ab proteins [20]. Starr et al. investigated the mutational space of SARS-CoV-2, and assays have been performed to measure the effect of spike protein mutations on binding to the ACE-2 receptor [21]. Suddnapas et al. studied the potential for LC-MS/MS identification of Beta, Gamma, Delta and Omicron variants based on synthetic peptides that mimic the unique tryptic peptides theoretically obtained from tryptic digestion of variants. This study evaluates the analytical method’s sensitivity for synthetic theoretical variant peptides, however this approach was not examined using cell-cultured SARS-CoV-2 variants. Variant detection is not only dependent on the prototypic peptides’ properties but also on the efficiency of variant tryptic digestion process and the highly complex background noise from the matrix of the cell-cultured virus [22]. Considering the current and probable future state of the growing repertoire of SARS-CoV-2 protein mutations, a method combining the universal detection of SARS-CoV-2 with the specific identification of SARS-CoV-2 variants would be valuable.

Here, we present an MS-based methodology for the diagnosis of SARS-CoV-2 that enables universal detection, independent of genetic mutations, alongside variant-specific identification. The universal detection of all SARS-CoV-2 variants is based on markers common to all variants, while the identification of the specific variant relies on variant-specific markers. Determining a specific set of markers for a given variant combines a multistep procedure starting with a search for in-silico derived variant tryptic peptides originating from viral proteins, followed by tryptic digest of a cell-cultured SARS-CoV-2 variant and HR-LC-MS/MS analysis of variant-specific peptides. This methodology can be further implemented for future SARS-CoV-2 variants or other emerging pathogens.

## 2. Materials and Methods

### 2.1. Reagents

Solvents and chemicals used in this study included: acetonitrile, water and formic acid (Bio Lab, Jerusalem, Israel), ammonium bicarbonate and Octyl-β-D-glucopyranoside (Sigma-Aldrich, Rehovot, Israel), phosphate-buffered saline and sequencing grade modified trypsin (Biological Industries, Beit Haemek, Israel). Cat. Numbers were previously reported [16].

### 2.2. Cell Lines and Viruses

African green monkey kidney clone E6 cells (Vero E6, ATCC^®^ CRL-1586™) were grown in Dulbecco’s modified Eagle’s medium (DMEM) containing 10% Fetal bovine serum (FBS), MEM nonessential amino acids (NEAA), 2 mM L-Glutamine, 100 Units/mL penicillin, 0.1 mg/mL streptomycin, 12.5 Units/mL Nystatin (P/S/N) (Biological Industries, Israel). Calu3 cells (ATCC HTB-55) were grown in RPMI supplemented with 10% FBS, NEAA, 2 mM L-glutamine, P/S/N, and 1% Na-pyruvate. Cells were cultured at 37 °C, in a 5% CO_2_ in 95% humidity.

SARS-CoV-2 wild-type strain (GISAID accession EPI_ISL_406862) was propagated (four passages) in Vero E6 cells. SARS-CoV-2 variants were provided by the Central Virology Lab of the Israel Ministry of Health [23]. Alpha (B.1.1.7, GISAID accession EPI_ISL_4169857) was passaged once in Vero E6, followed by two passages in Calu3 cells. Beta (B.1.351, GISAID accession EPI_ISL_4169885), Gamma (P.1, GISAID accession EPI_ISL_4169886) and Delta (B.1.617.2, GISAID accession EPI_ISL_4169986) variants were propagated in Vero E6 cells. Virus culturing was conducted in a BSL3 facility according to the biosafety guidelines of the Israel Institute for Biological Research (IIBR). Virus stock titration was performed on Vero E6 cells as described [16].

### 2.3. Tryptic Digestion

An efficient tryptic digestion process was performed as previously described. Briefly, samples in a volume of 100 µL (PBS spiked with 10^6^ pfu/mL SARS-CoV-2 variants) were heat-denatured (95 °C, 10 min) in the presence of 0.2% Octyl β Glucopyranoside. After cooling, 2 µL of sequencing grade modified trypsin (0.5 μg/µL) were added (final concentration 1 μg/100 µL), followed by 2 h incubation at 50 °C with continuous rotation (600 RPM). Tryptic digestion was stopped as described [16,17].

### 2.4. High-Resolution LC–MS/MS (Orbitrap)

LC-MS analysis was performed on an Agilent 1290 HPLC (Agilent Technologies, Palo Alto, CA, USA) coupled to Q-exactive plus Orbitrap MS/MS instrument (Thermo Fisher Scientific, Waltham, MA, USA) equipped with a heated electrospray ionization source operated in positive mode. This high-resolution MS system enables the identification of SARS-CoV-2 proteins derived from genetic variants’ tryptic peptides according to their accurate mass and sequence determination using Full MS DIA acquisition mode. Chromatographic separations were performed on a 1.7 µm UPLC C18 column (150 mm × 2.1 mm, 1.7 µm) kept at 40 °C, based on charged surface hybrid (CSH) technology, applying water/acetonitrile acidic (1% formic acid) gradient and a 10 min cycle time. Mobile phases were 1% formic acid in H_2_O (A) and 1% formic acid in ACN:H_2_O (4:1 *v*/*v*, B). The gradient profile was 100% A held for 0.3 min, linearly decreased to 75% A over 4 min, held for 0.5 min, then decreased to 0% A over 2.5 min, held for 1 min, then increased to 100% A over 0.1 min and held for another 1.9 min, for a total run time of 10 min. The flow rate was 0.4 mL/min and the injection volume was 10 µL. The operating parameters were as previously described [16].

### 2.5. Bioinformatic Analysis

The list of mutations in the Alpha, Beta, Gamma and Delta variants with regard to the SARS-CoV-2 reference sequence (Wuhan, accession # NC_045512.2) was generated from variant-call analysis of the in-house whole genome sequencing conducted in the past and submitted to the GISAID depository (GISAID identifiers as follows: Alpha variant: EPI_ISL_416985, Beta variant: EPI_ISL_41669885, Gamma variant: EPI_ISL_4169886, Delta variant: EPI_ISL_4169986). The mutations detected were all in accordance with the documented list of mutations available at the time of the research (Alpha strain [24], Beta strain [25], Gamma strain [26], Delta strain www.outbreak.info.com, accessed on 17 August 2021). The list of mutations in each of the four variants, as mapped from the in-house genomic sequencing, is provided in Table S1. Prediction of potential trypsin cleavage sites was conducted with PeptideCutter (www.web.expasy.org/peptide_cutter, accessed on 25 October 2021). The resulted peptides were analyzed against the nr database (https://www.ncbi.nlm.nih.gov/, accessed on 15 November 2021) using Blast [27]. The algorithm parameters were adjusted to short sequences and self-hits were eliminated.

## 3. Results and Discussion

### 3.1. Methodology for Specific SARS-CoV-2 Variant Diagnosis

**Figure 1:**
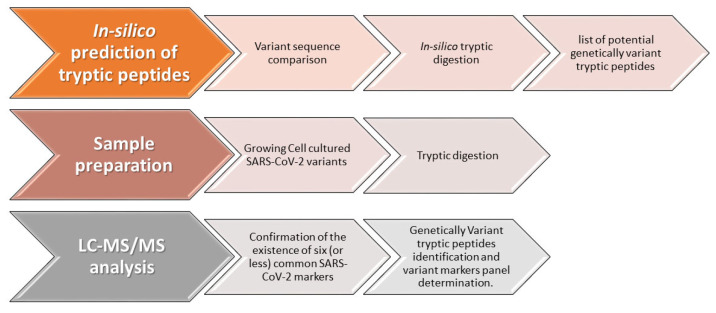
Methodology for developing MS-based diagnosis of SARS-CoV-2 variants.

Recently we have published an assay for COVID-19 diagnosis based on identification of six unique and specific peptide markers derived from the SARS-CoV-2 spike and nucleocapsid proteins [16,17]. The sequence conservation of these markers, among different known strains of SARS-CoV-2 was of major concern, considering the accumulating knowledge regarding mutations occurring during the spread of the virus. Therefore, a main parameter in the selection of these markers was their universality (identical sequence in >99.8% of known strains). However, for discriminative identification of SARS-CoV-2 VOCs, the identification of unique variant markers is required. Nucleotide polymorphisms resulting in peptide variations are characterized by specific amino acids substitutions, which determine variants marker unique sequences. Our new methodology for developing a variant-specific diagnostic method is schematically shown in Figure 1. Defining a specific set of markers for a given variant combines a multistep procedure starting with in-silico identification of trypsin-digested peptides originating from viral proteins, followed by tryptic digest of a cell-cultured SARS-CoV-2 variant and HR-LC-MS/MS analysis for peptides identification and variant markers panel determination. The final method is an expanding analytical method for identification of SARS-CoV-2 variants, which is based on a fixed panel of markers common to all variants and on a flexible set of variant-specific markers which is updated for newly discovered variants. Given a known variant, the universal markers shared by all variant strains and those specific to that variant will be identified in the specimen. For a new emerging variant, only the markers common to all variants will be detected in the sample. Knowing the sequence of a new variant and using our methodology for developing MS-based diagnosis of SARS-CoV-2 variants will enable adding a set of variant-specific markers to the expanding analytical method.

### 3.2. In-silico Mapping of Potential Variant-Specific Markers

As mentioned, our aim was to evaluate peptides that may provide a diagnostic tool for variant identification using MS. We therefore first conducted an in-silico tryptic digestion analysis of the viral proteins containing mutations compared to the Wuhan reference strain (Table S1). This analysis was conducted for four representative VOCs—Alpha, Beta, Gamma and Delta. Each peptide generated by the in-silico tryptic digest and containing a mutation with respect to its orthologous sequence in the Wuhan reference sequence, was considered as a potential candidate. Only peptides longer than five amino acids were considered, to minimalized cross-identification with unrelated, non-SARS-CoV-2 proteins, possibly leading to false positive results. To avoid adsorption of the peptides to the chromatographic system, which may reduce the assay’s sensitivity, we focused on peptides that were shorter than 24 amino acids. The in-silico analysis yielded a total of 37 potential peptide markers (6, 6, 13 and 12 for the Alpha, Beta, Gamma and Delta variants, respectively) (Table S2), of which almost half were derived from the highly abundant spike protein. To further assess their specificity with respect to other human pathogens, the peptides were subjected to a sequence similarity search against the comprehensive NCBI non-redundant (nr) database, revealing 28 unique peptides, and 9 additional peptides that share an ortholog with a non-relevant human clinical organism (Table S2).

### 3.3. Identification of Unique Markers in Cell-Cultured VOCs Using HR-LC-MS/MS Analysis

To evaluate the actual ability to identify the predicted variant-specific markers and their formation in real life samples, we cultured the four variants (Alpha, Beta, Gamma, and Delta) in Vero E6 cells. A rapid, simple, and efficient tryptic digestion process was performed, similar to the procedure utilized in our previous studies [16,17]. Samples containing 10^6^ PFU/mL of each cell-cultured SARS-CoV-2 variant were preheated (95 °C, 10 min) to inactivate the virus and denature viral proteins, which improved subsequent digestion efficiency. The samples were then trypsin-digested (in triplicates, 120 min 50 °C) followed by analysis using LC-coupled to a high-resolution MS/MS.

The analytical method described recently [16,17] was used for the identification of SARS-CoV-2 variants peptides markers. First, the existence of the six universal markers was proven to be common to all SARS-CoV-2 variants. To characterize a unique set of markers for each variant (Alpha, Beta, Gamma, and Delta), the potential markers were searched according to the *m/z* of multiple charged ions which were calculated for the in-silico predicted tryptic peptides (Table S2). For each variant, at least two unique markers, derived mainly from S and N viral proteins, were identified using LC-MS/MS analysis, according to accurate mass and fragmentation pattern, which indicate the amino acid sequences (Table 1). These markers appeared in all replicates for each variant. The characteristic markers of each variant were not found in any of the other variants, ascertaining their uniqueness to the specific variant strain.

**Figure 2:**
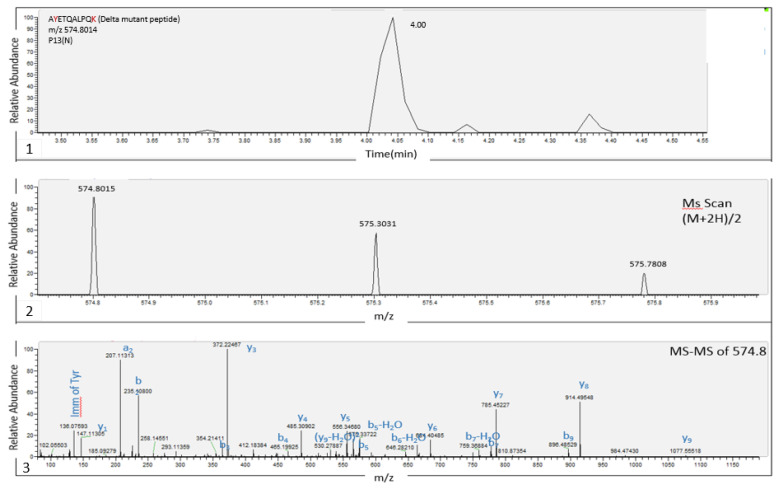
LC-MS (Orbitrap) analysis of cell-cultured SARS-CoV-2 variants after tryptic digestion. LC-MS/MS identification of SARS-CoV-2 Delta variant-specific marker, A**Y**ETQALPQ**K**. (1) Extracted-ion Chromatograms (EIC) of *m*/*z* 574.8014 from a full scan LC-MS run of Cell-Cultured SARS-CoV-2 (10^6^ PFU/mL) Delta variant. (2) Mass spectrum of the specific marker A**Y**ETQALPQ**K** (parent ion, (M + 2H)/2, at *m*/*z* 574.8014, chromatographic peak at 4.04 min) derived from SARS-CoV-2 Delta variant. (3) Marker fragmentation spectrum (MS-MS of 574.8).

**Figure 3:**
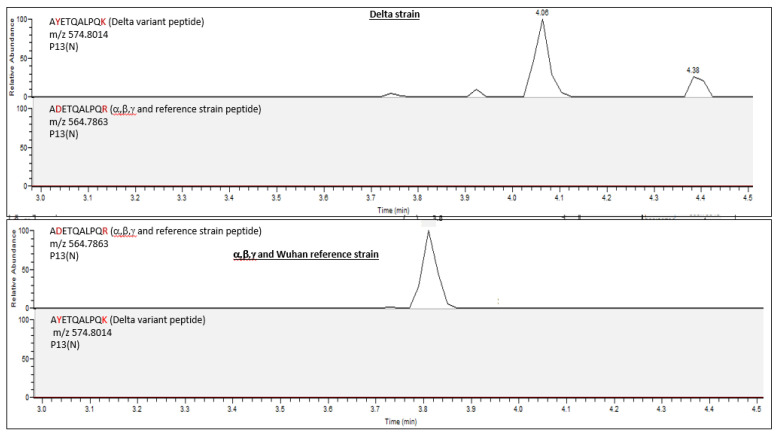
An Extracted-ion Chromatogram (EIC) of Delta variant-specific marker (mutant peptide, which was derived from nucleocapsid protein, p13(N), A**Y**ETQALPQ**K**). It was not detected in other variants, while the corresponding wild-type peptide (prior to mutation) (A**D**ETQALPQ**R**) was not detected in Delta variant but was detected in all other variants.

**Figure 4:**
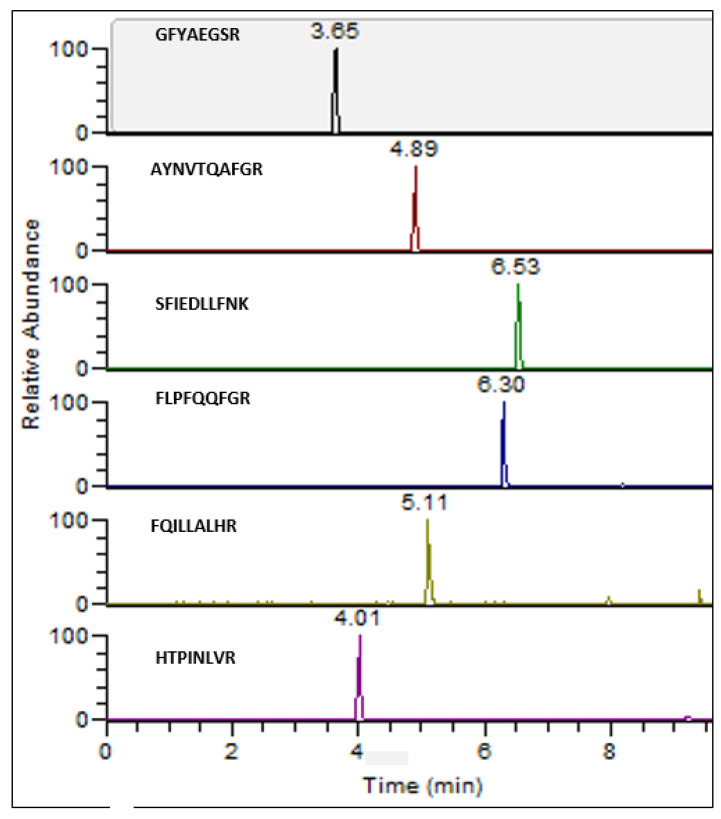
Extracted-ion Chromatograms (EIC) of six common markers to all SARS-CoV-2 variants besides S-18 marker that was not detected in Beta variant.

Figure 2, Figure 3 and Figure 4 demonstrates the analysis of cell-cultured SARS-CoV-2 variants after tryptic digestion. A chromatographic peak, eluted at 4 min as a double charged ion (M+2H)/2 at *m/z* 574.8014, belongs to representative unique marker of the Delta variant, A**Y**ETQALPQ**K**, in which the amino acids Y and K represent mutations of D and R, respectively (A**D**ETQALPQ**R**). It’s fragmentation spectrum is presented below.

This marker was not detected in the wild-type Wuhan reference strain nor in any other VOC. In concordance with these findings, the corresponding wild-type peptide, A**D**ETQALPQ**R**, was detected in all other VOCs but not in the Delta variant (Figure 3).

The six SARS-CoV-2 universal markers were indeed found in the Delta variant as well as in all the other analyzed variants (Figure 4).

### 3.4. Discussion and Conclusions

In this study we suggest an expanded method to monitor and specifically identify SARS-CoV-2 variants. This method combines the analysis of SARS-CoV-2 universal markers characterized by relatively high sequence conservation, resulting in a high likelihood of being shared by all variants, together with specific markers for each variant. This novel approach for discriminative identification based on MS analysis is presented and demonstrated for four representative SARS-CoV-2 VOCs (Alpha, Beta, Gamma, and Delta). This methodology is based on a multistep procedure starting with prediction of in-silico derived tryptic digested peptides originating from viral proteins, followed by actual tryptic digest of a cell-cultured SARS-CoV-2 variant and HR-LC-MS/MS analysis for variant markers panel determination. The samples used in this study were cell-cultured viruses (10^6^ pfu/mL). Previous studies demonstrated the ability to identify virus-specific markers directly from nasopharyngeal clinical samples or after pre-concentration and purification step using immunomagnetic beads, with LODs of 5 × 10^4^ or 1 × 10^3^ pfu/mL, respectively. The proposed methodology has two main limitations: (1) virus cultivation had to be conducted in a BSL3 facility, in accordance with the biosafety guidelines and (2) this method necessitates preliminary knowledge of the variant sequence that can be achieved only through sequence analysis. On the other hand, the proposed methodology precludes the subsequent time-consuming and relatively expensive sequencing of further samples for diagnostic purposes. This procedure, compared to genetic sequencing, is rapid (taking only a few hours), easy to perform and provides an inexpensive specific diagnosis method for SARS-CoV-2 variants. The methodology demonstrated in this study, as a proof of concept, may be adjusted and implemented for the diagnosis of other SARS-CoV-2 variants or for a variety of other pathogens as well.

## Figures and Tables

**Table 1:** Unique markers for SARS-CoV-2 variants identified by High resolution LC-MS/MS (Orbitrap).

Variant	Unique Markers	Viral Protein	Parent Ions
**Alpha**	VCEFQFCNDPFLGVYHK	P1-Spike	682.6469 (M+3H)/3
	IFTIGTVTFK	P9-Orf3a	563.8295 (M+2H)/2
**Beta**	GISPAR	P1-N	600.3469 (M+H), 300.6773 (M+2H)/2
	QIAPGQTGNIAQYNYK	P5-Spike	876.9317 (M+2H)/2
**Gamma**	GQGVPINTNSSR	P14-N	615.3158 (M+2H)/2
	QIAPGQTGTIADYNYK	P8-Spike	870.4341 (M+2H)/2
	ASANLAAIK	P12-Spike	429.7563 (M+2H)/2
	TQLPSAYTNSFTR	P5-Spike	743.3707 (M+2H)/2
**Delta**	QIAPGQTGTIADYNYK	P8-Spike	870.4341 (M+2H)/2
	ASANLAAIK	P12-Spike	429.7563 (M+2H)/2
	TQLPSAYTNSFTR	P5-Spike	743.3707 (M+2H)/2
	VGGNYNYR	P7-Spike	471.7255 (M+2H)/2

## Data Availability

Data is contained within the article or Supplementary Materials.

## Supplementary Materials

The following supporting information can be downloaded at: https://www.mdpi.com/article/10.3390/biomedicines11092373/s1, Table S1: List of mutations with regard to the Wuhan reference strain; Table S2: Potential peptide markers for the Alpha, Beta, Gamma, and Delta SARS-CoV-2 variants; Table S3: Unique markers for SARS-CoV-2 variants identified by High Resolution LC-MS/MS (Orbitrap).
