# Supplementary material for: Discriminative Identification of SARS-CoV-2 Variants Based on Mass-Spectrometry Analysis
Source: Biomedicines. 2023 Aug 24;11(9):2373. doi: 10.3390/biomedicines11092373 (PMC10525290; doi:10.3390/biomedicines11092373)
Supplement: Supplementary file 1 [file biomedicines-11-02373-s001.zip › Table S1.pdf]

Table S1: List of mutations with regard to the Wuhan reference strain

	UK	SA	BR	IN
**Orf1ab**	T1001I	T265I	S1188L	P314L
	A1708D	P1220L	K1795Q	G662S
	I2230T	K1655N	S2553F	P1000L
	F2780L	K3353R	del3675-3677 (SGF)	H2285Y
	del 3675-3677 (SGF)	del3675-3677 (SGF)	L3829F	
	L3829F	L3829F	E5665D	
	P4715L	P4715L		
**Spike**	del69-70 (HV)	L18F	L18F	T19R
	del144(Y)	D80A	T20N	G142D
	N501Y	D215G	P26S	del 157/158
	A570D	del241-243* (LLA)	D138Y	L452R
	P681H	K417N	R190S	T478K
	T716I	E484K	K417T	D614G
	S982A	N501Y	E484K	P681R
	D1118H	R682L	N501Y	R682W
		A701V	H655Y	D950N
			T1027I	
			V1176F	
**Orf3a**		Q57H		S26L
		S171L		
**E**		P71L		
**M**				I82T
**Orf7a**				V82A
				L116F
				T120I
**Orf8**	Q27 stop		E92K	del119/120
	R52I		Ins28269-28273	
	Y73C			
**N**	D3L	T205I	P80R	D63G
	203-204 (RG>KR)		203-204 (RG>KR)	R203M
	S235F			D377Y
				R385K
