# Supplementary material for: Discriminative Identification of SARS-CoV-2 Variants Based on Mass-Spectrometry Analysis
Source: Biomedicines. 2023 Aug 24;11(9):2373. doi: 10.3390/biomedicines11092373 (PMC10525290; doi:10.3390/biomedicines11092373)
Supplement: Supplementary file 1 [file biomedicines-11-02373-s001.zip › Table S2.pptx]

## Slide 1
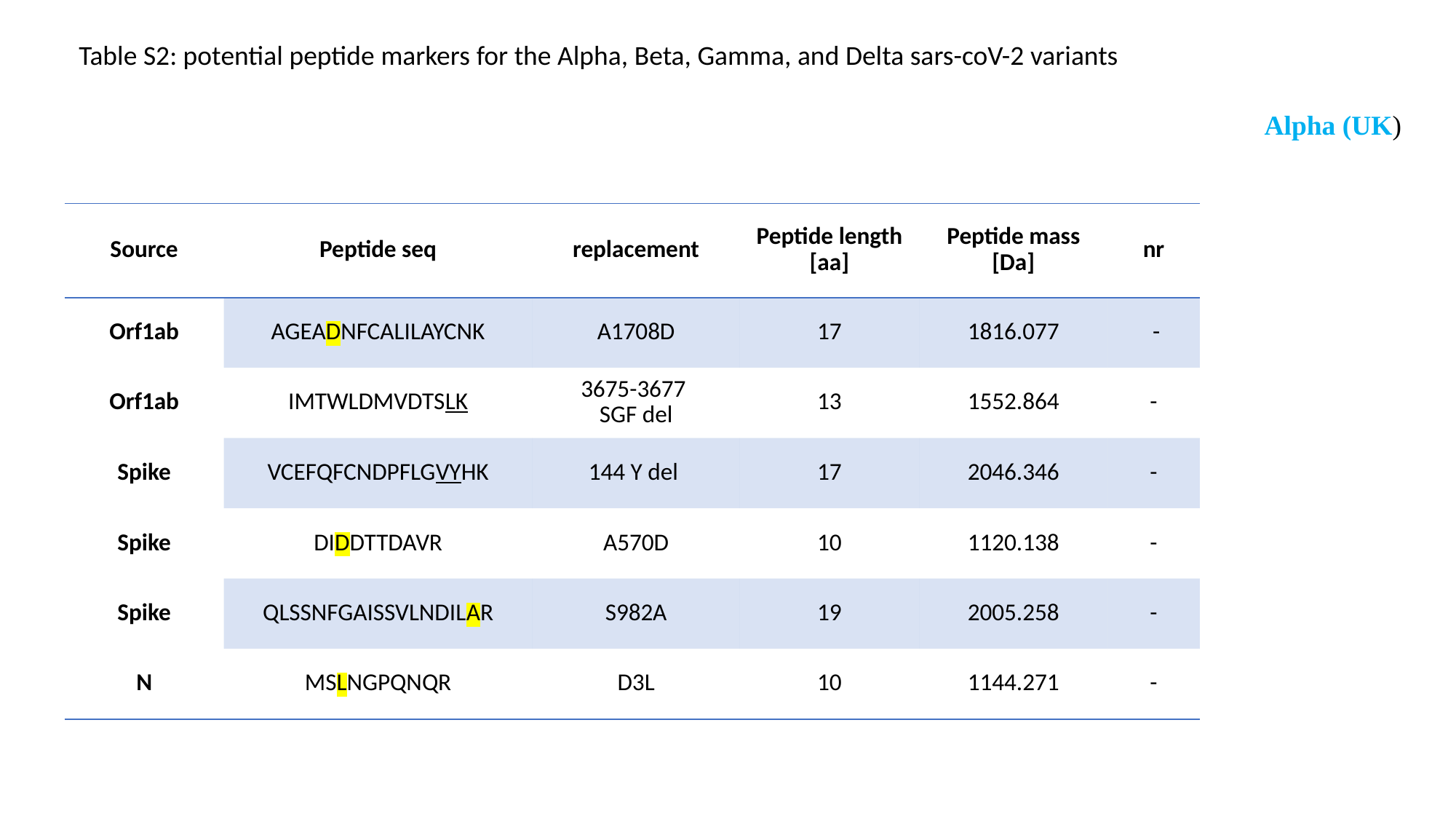

Table S2: potential peptide markers for the Alpha, Beta, Gamma, and Delta sars-coV-2 variants
Alpha (UK)
| Source | Peptide seq | replacement | Peptide length [aa] | Peptide mass [Da] | nr |
| --- | --- | --- | --- | --- | --- |
| Orf1ab | AGEADNFCALILAYCNK | A1708D | 17 | 1816.077 | - |
| Orf1ab | IMTWLDMVDTSLK | 3675-3677 SGF del | 13 | 1552.864 | - |
| Spike | VCEFQFCNDPFLGVYHK | 144 Y del | 17 | 2046.346 | - |
| Spike | DIDDTTDAVR | A570D | 10 | 1120.138 | - |
| Spike | QLSSNFGAISSVLNDILAR | S982A | 19 | 2005.258 | - |
| N | MSLNGPQNQR | D3L | 10 | 1144.271 | - |

## Slide 2
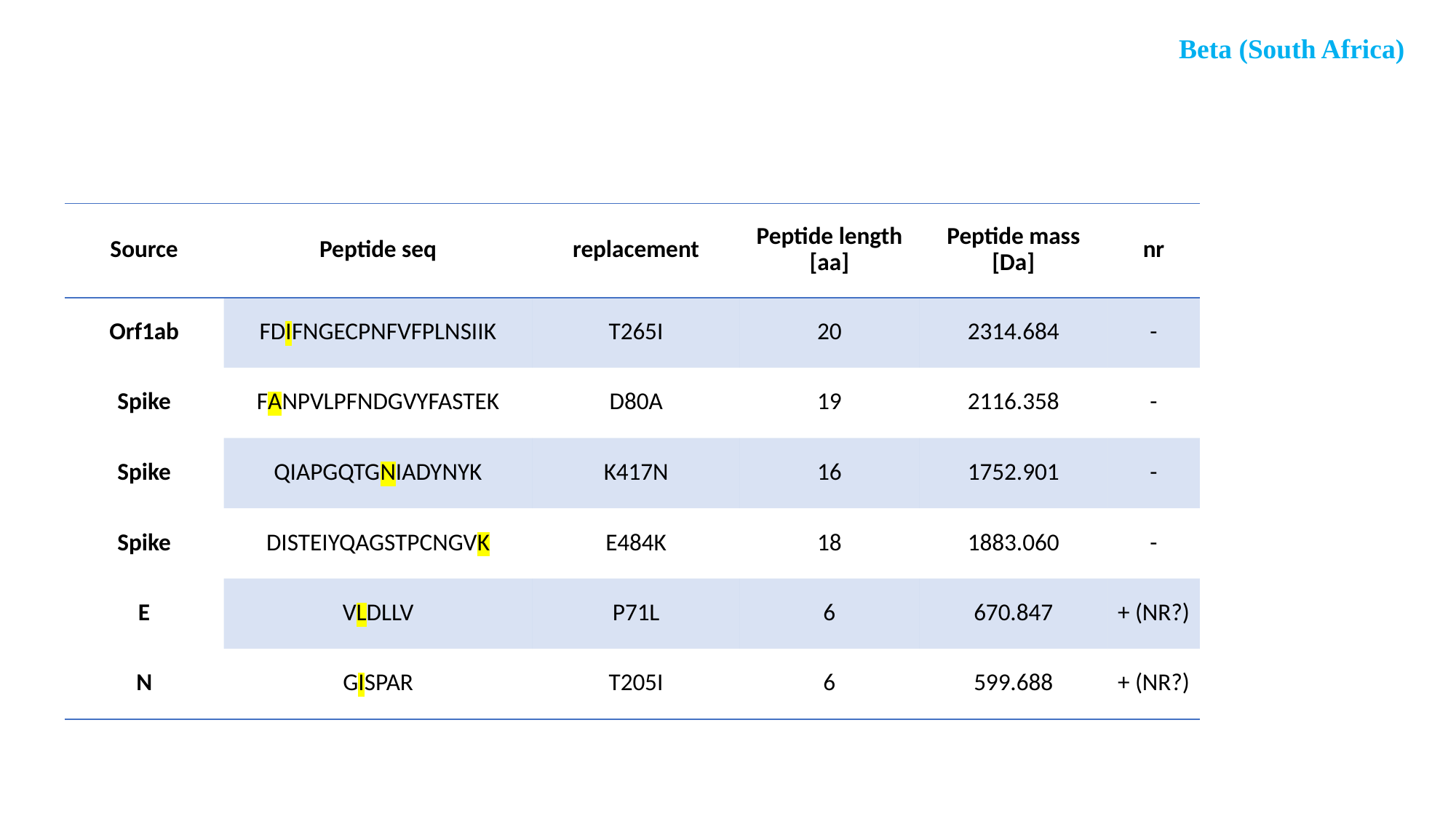

Beta (South Africa)
| Source | Peptide seq | replacement | Peptide length [aa] | Peptide mass [Da] | nr |
| --- | --- | --- | --- | --- | --- |
| Orf1ab | FDIFNGECPNFVFPLNSIIK | T265I | 20 | 2314.684 | - |
| Spike | FANPVLPFNDGVYFASTEK | D80A | 19 | 2116.358 | - |
| Spike | QIAPGQTGNIADYNYK | K417N | 16 | 1752.901 | - |
| Spike | DISTEIYQAGSTPCNGVK | E484K | 18 | 1883.060 | - |
| E | VLDLLV | P71L | 6 | 670.847 | + (NR?) |
| N | GISPAR | T205I | 6 | 599.688 | + (NR?) |

## Slide 3
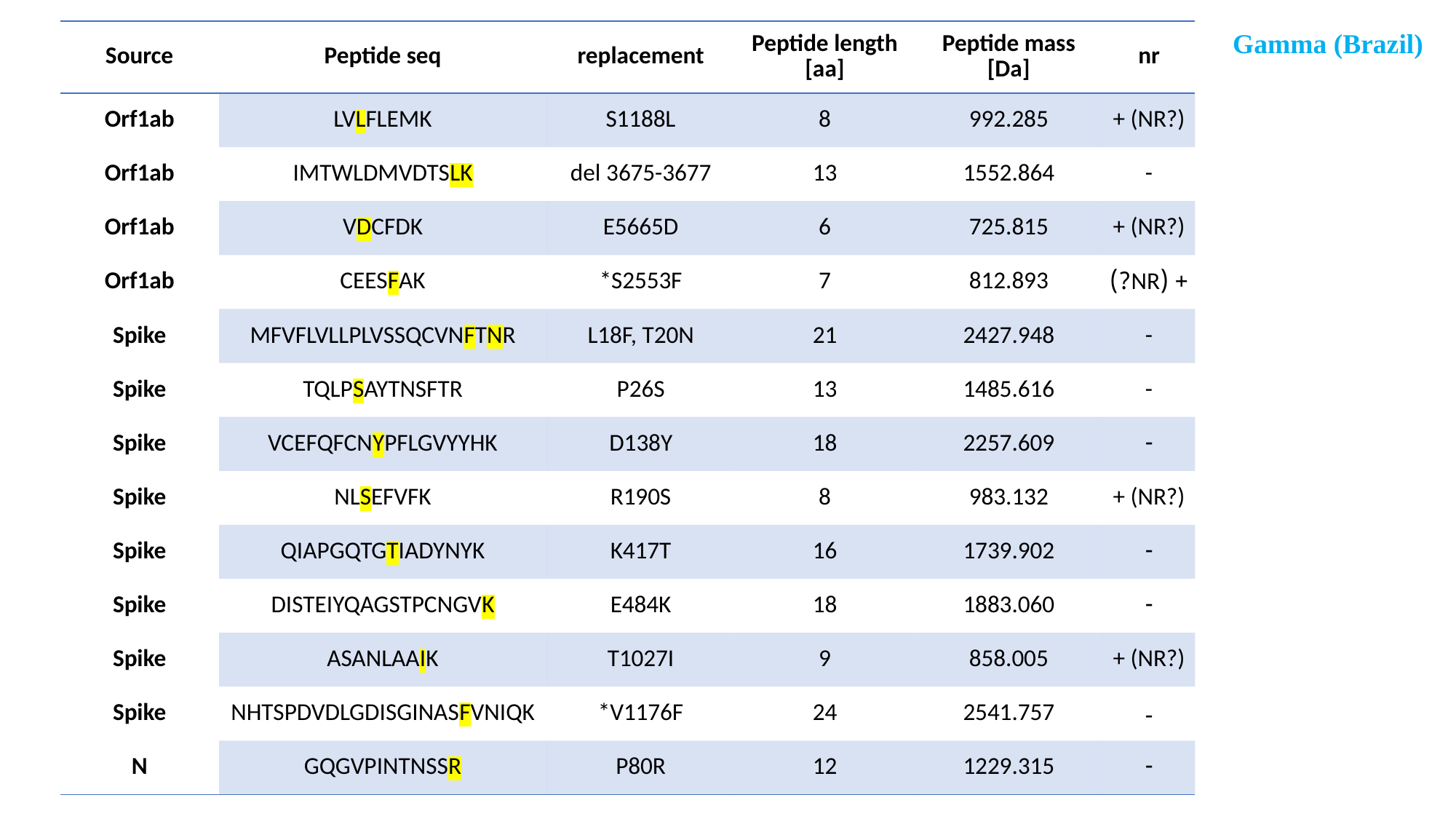

| Source | Peptide seq | replacement | Peptide length [aa] | Peptide mass [Da] | nr |
| --- | --- | --- | --- | --- | --- |
| Orf1ab | LVLFLEMK | S1188L | 8 | 992.285 | + (NR?) |
| Orf1ab | IMTWLDMVDTSLK | del 3675-3677 | 13 | 1552.864 | - |
| Orf1ab | VDCFDK | E5665D | 6 | 725.815 | + (NR?) |
| Orf1ab | CEESFAK | \*S2553F | 7 | 812.893 | + (NR?) |
| Spike | MFVFLVLLPLVSSQCVNFTNR | L18F, T20N | 21 | 2427.948 | - |
| Spike | TQLPSAYTNSFTR | P26S | 13 | 1485.616 | - |
| Spike | VCEFQFCNYPFLGVYYHK | D138Y | 18 | 2257.609 | - |
| Spike | NLSEFVFK | R190S | 8 | 983.132 | + (NR?) |
| Spike | QIAPGQTGTIADYNYK | K417T | 16 | 1739.902 | - |
| Spike | DISTEIYQAGSTPCNGVK | E484K | 18 | 1883.060 | - |
| Spike | ASANLAAIK | T1027I | 9 | 858.005 | + (NR?) |
| Spike | NHTSPDVDLGDISGINASFVNIQK | \*V1176F | 24 | 2541.757 | - |
| N | GQGVPINTNSSR | P80R | 12 | 1229.315 | - |
Gamma (Brazil)

## Slide 4
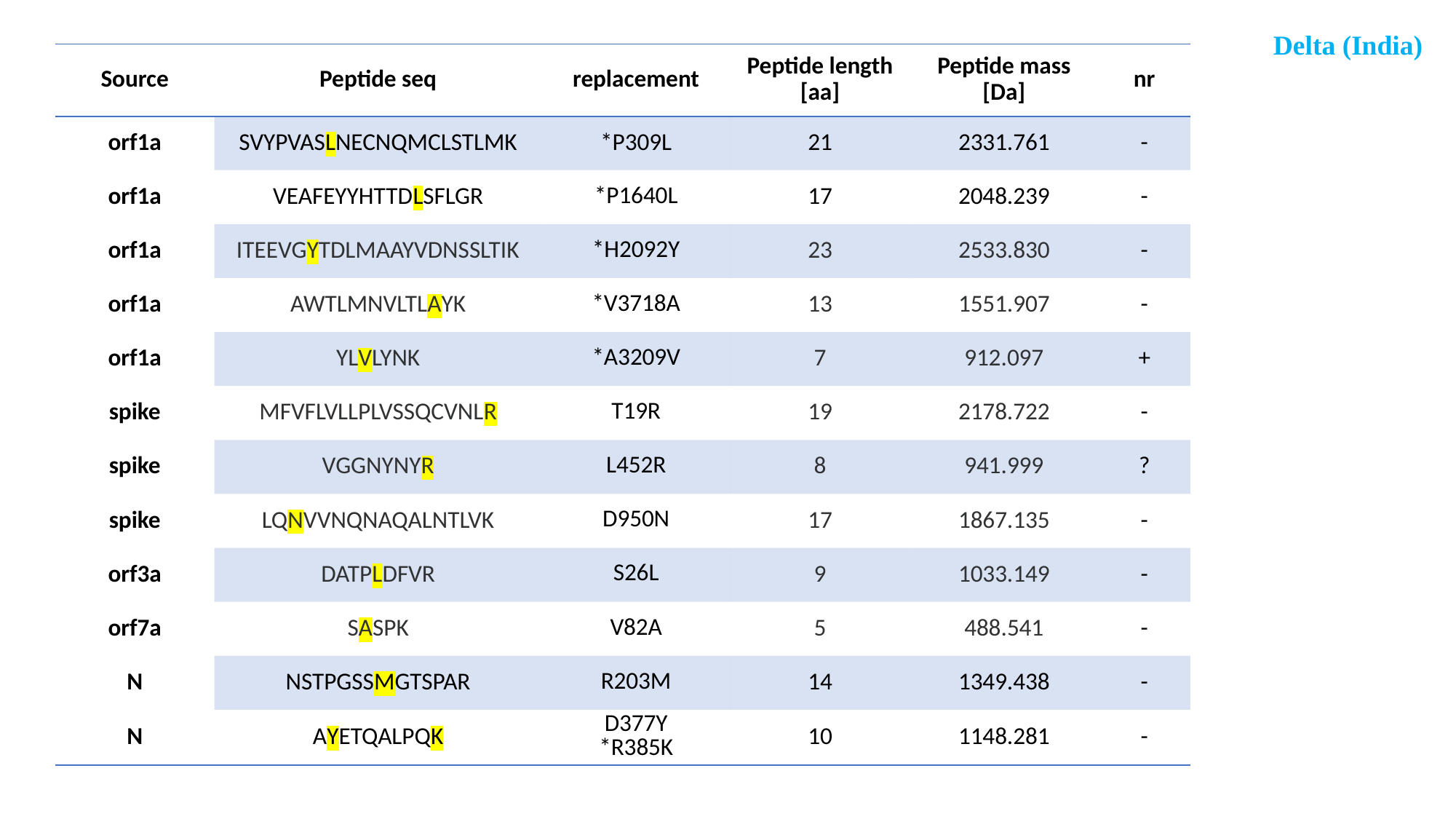

Delta (India)
| Source | Peptide seq | replacement | Peptide length [aa] | Peptide mass [Da] | nr |
| --- | --- | --- | --- | --- | --- |
| orf1a | SVYPVASLNECNQMCLSTLMK | \*P309L | 21 | 2331.761 | - |
| orf1a | VEAFEYYHTTDLSFLGR | \*P1640L | 17 | 2048.239 | - |
| orf1a | ITEEVGYTDLMAAYVDNSSLTIK | \*H2092Y | 23 | 2533.830 | - |
| orf1a | AWTLMNVLTLAYK | \*V3718A | 13 | 1551.907 | - |
| orf1a | YLVLYNK | \*A3209V | 7 | 912.097 | + |
| spike | MFVFLVLLPLVSSQCVNLR | T19R | 19 | 2178.722 | - |
| spike | VGGNYNYR | L452R | 8 | 941.999 | ? |
| spike | LQNVVNQNAQALNTLVK | D950N | 17 | 1867.135 | - |
| orf3a | DATPLDFVR | S26L | 9 | 1033.149 | - |
| orf7a | SASPK | V82A | 5 | 488.541 | - |
| N | NSTPGSSMGTSPAR | R203M | 14 | 1349.438 | - |
| N | AYETQALPQK | D377Y \*R385K | 10 | 1148.281 | - |
