# Supplementary material for: Discriminative Identification of SARS-CoV-2 Variants Based on Mass-Spectrometry Analysis
Source: Biomedicines. 2023 Aug 24;11(9):2373. doi: 10.3390/biomedicines11092373 (PMC10525290; doi:10.3390/biomedicines11092373)
Supplement: Supplementary file 1 [file biomedicines-11-02373-s001.zip › Table S3.pdf]

Table S3: Unique markers for SARS-Cov-2 variants identified by High resolution LC-MS/MS (Orbitrap)

variant	Unique Markers	Parent ions	r.t	MS-MS fragments
Alpha (UK)	VCEFQFCNDPFLGVYHK (P1-Spike)	682.6469 (M+3H)/3	6.5	284.172 $(y_2)^{+1}$, 447.236 $(y_3)^{+1}$, 546.304 $(y_4)^{+1}$, 603.325 $(y_5)^{+1}$, 716.409 $(y_6)^{+1}$, 863.478 $(y_7)^{+1}$, 960.531 $(y_8)^{+1}$, 302.167 $(y_5)^{+2}$, 358.709 $(y_6)^{+2}$, 432.243 $(y_7)^{+2}$, 480.769 $(y_8)^{+2}$
	IFTIGTVTFK (P9-Orf3a)	563.8295 (M+2H)/2	6.5	86.097 (imm of Ile), 120.081 (imm of Phe), 147.172 $(y_1)^{+1}$, 294.182 $(y_2)^{+1}$, , 652.367 $(y_6)^{+1}$, 765.451 $(y_7)^{+1}$, 866.499 $(y_8)^{+1}$, 1013.567 $(y_9)^{+1}$, 995.557 $(y_9 – H_2O)^{+1}$, 261.160 $(b_2)^{+1}$, 362.208 $(b_3)^{+1}$, 507.288 $(y_9)^{+2}$
Beta (South Africa)	GISPAR (P1-N)	600.3469 (M+H) 300.6773 (M+2H)/2	3.2	430.241$(y_4)^{+1}$, 343.209 $(y_3)^{+1}$, 246.157 $(y_2)^{+1}$, 175.119 $(y_1)^{+1}$, 413.215 $(z_4)^{+1}$, 326.183 $(z_3)^{+1}$, 412.231 $(y_4\text{-}H_2O)^{+1}$, 325.199 $(y_3\text{-}H_2O)^{+1}$, 413.215 $(y_4\text{-}NH_3)^{+1}$, 395.204 $(Z_4\text{-}H_2O)^{+1}$, 171.113 (b2), 258.145 (b3), 355.198 (b4), 143.118 (a4), 240.135 $(b_3\text{-}H_2O)^+$, 355.198 $(c_3\text{- }NH_3)^+$, 272.167 $(y_5)^{+2}$, 215.625 $(y_4)^{+2}$,172.109 $(y_3)^{+2}$, 86.097 (Imm of Ile), 60.045 (Imm of Ser)
	QIAPGQTGNIAQYNYK (P5-Spike)	876.9317 (M+2H)/2	4.8	1511.558 $(y_{14})^{+1}$, 1440.676 $(y_{13})^{+1}$, 1158.543 $(y_{10})^{+1}$, 1057.495 $(y_9)^{+1}$, 773.347 $(y_6)^{+1,}$ 702.310 $(y_5)^{+1}$, 587.283 $(y_4)^{+1}$, 424.283 $(y_4)^{+1}$ 129.066 $(b_1)^{+1}$, 242.150 $(b_2)^{+1}$, 313.188 $(b_3)^{+1}$, 756.360 $(y_{14})^{+2}$ 720.842 $(y_{13})^{+2}$
Delta (India)	AYETQALPQK P13(N)	574.8014 (M+2H)/2	4.0	1077.558 $(y_9)^{+1}$, 914.495 $(y_8)^{+1}$, 785.452 $(y_7)^{+1}$, 684.404 $(y_6)^{+1}$, 556.346 $(y_5)^{+1}$, 485.309 $(y_4)^{+1}$, 372.225 $(y_3)^{+1}$, 275.172 $(y_2)^{+1}$, 147.113 $(y_1)^{+1}$, 235.108 $(b_2\text{-}H_2O)^{+1}$, 207.113 $(a_2)^{+1}$, 759.368 $(b_7\text{-}H_2O)^+$, 646.284 $(b_6\text{-}H_2O)^{+1}$, 575.247 $(b_5\text{-}H_2O)^{+1,}$ 447.188 $(b_4\text{-}H_2O)^{+1}$ 136.076 (Imm of Tyr), 984.479 $(b_9)^{+1}$, 777.378 $(b_7)^{+1}$, 749.383 $(a_7)^{+1}$, 732.357 $(a_7\text{-}NH_3)^{+1}$, 667.378 (Z_6), 593.257 $(b_5)^{+1}$, 539.283 $(y_9)^{+2}$, 530.278 $(y_9\text{-}H_2O)^{+2}$, 393.230 $(y_7)^{+2}$, 465.198 $(b_4)^{+1}$, 457.751 $(y_8)^{+2}$, 420.177 $(a_4\text{-}NH_3)^+$
	LQNVVNQNAQALNTLVK P8 (Spike)	934.0239 (M+2H)/2 623.0185 (M+3H)/3	5.9	1412.786 $(y_{13})^{+1}$, 1313.718 $(y_{12})^{+1}$, 1071.616 $(y_{10})^{+1}$, 957.573 $(y_9)^{+1}$, 886.536 $(y_8)^{+1}$, 758.478 $(y_7)^{+1}$, 574.356 $(y_5)^{+1}$, 460.314 $(y_4)^{+1}$, 706.897 $(y_{13})^{+2}$, 443.772 $(y_8)^{+2}$, 242.150 $(b_2)^{+1}$, 356.193 $(b_3)^{+1}$, 455.262 $(b_4)^{+1}$, 554.330 $(b_5)^{+1}$, 796.432 $(b_7)^{+1}$, 981.512 $(b_9)^{+1}$, 228.135 $(b_4)^{+2}$, 277.669 $(b_5)^{+2}$, 344.690 $(b_6)^{+2}$, 398.720 $(b_7)^{+2}$, 455.741 $(b_8)^{+2, 491.260}$ $(b_9)^{+2}$, 555.289 $(b_{10})^{+2}$
	NSTPGSSMGTSPAR P12 (N)	675.3098 (M+2H)/2	3.7	1148.537 $(y_{12})^{+1}$, 1047.489 $(y_{11})^{+1}$, 950.437 $(y_{10})^{+1}$, 863.415 $(y_9)^{+1}$, 806.383 $(y_8)^{+1}$, 719.351 $(y_7)^{+1}$, 588.311 $(y_6)^{+1}$, 430.241 $(y_4)^{+1}$, 343.209 $(y_3)^{+1}$, 175.120 $(y_1)^{+1}$, 618.288 $(y_{13})^{+2}$, 574.772 $(y_{12})^{+2}$, 524.249 $(y_{11})^{+2}$, 202.083 $(b_2)^{+1}$, 303.130 $(b_3)^{+1}$
	VGGNYNYR P7 (Spike)	471.7255 (M+2H)/2	3.6	843.375 $(y_7)^{+1}$, 786.353 $(y_6)^{+1}$, 729.332 $(y_5)^{+1}$, 615.289 $(y_4)^{+1}$, 452.226 $(y_3)^{+1}$, 338.183 $(y_2)^{+1}$, 175.120 $(y_1)^{+1}$, 422.191 $(y_7)^{+2}$, 393.681 $(y_6)^{+2}$, 157.098 $(b_2)^{+1}$, 72.081 (Imm of Val), 136.076 (Imm of Tyr)
Gamma (Brazil)	GQGVPINTNSSR (P14-N)	615.3158 (M+2H)/2	3.9	186.088 $(b_2)^{+1}$, 169.061 ($b_2\text{-}NH_3$), 243.109 $(b_3)^{+1}$,314.1778 $(a_4)^{+1}$, 342.178$(b_4)^{+1}$, 296.172 $(a_4\text{-}H_2O)$, 411.236$(a_5)^{+1}$, 888.454$(y_8)^{+1}$, 871.427 $(z_8)^{+1}$, 791.401$(y_7)^{+1}$, 678.317$(y_6)^{+1}$, 494.265$(y_9)^{+2}$, 444.731$(y_8)^{+2}$
	QIAPGQTGTIADYNYK (P8-Spike)	870.4341 (M+2H)/2	4.8	1498.718$(y_{14})^{+1}$, 1427.681$(y_{13})^{+1}$, 1330.628$(y_{12})^{+1}$, 1145.548$(y_{10})^{+1}$, 1044.500$(y_9)^{+1}$, 987.479$(y_8)^{+1}$, 886.431$(y_7)^{+1}$, 773.347$(y_6)^{+1}$, 702.310$(y_5)^{+1}$, 587.283$(y_4)^{+1}$, 424.220$(y_3)^{+1}$, 310.177$(y_2)^{+1}$, 147.113$(y_1)^{+1}$, 242.150$(b_2)^{+1}$, 313.188$(b_3)^{+1}$, 595.320$(b_6)^{+1}$, 967.521$(b_{10})^{+1}$, 749.863$(y_{14})^{+2}$, 714.344$(y_{13})^{+2}$, 136.076 (Imm of Tyr)
	ASANLAAIK (P12-Spike)	429.7563 (M+2H)/2	4.1	787.468$(y_8)^{+1}$, 700.436$(y_7)^{+1}$, 629.399$(y_6)^{+1}$, 515.356$(y_5)^{+1}$, 402.272$(y_4)^{+1}$, 331.234$(y_3)^{+1}$, 260.197$(y_2)^{+1}$, 147.113$(y_1)^{+1}$, 159.077$(b_2)^{+1}$, 344.157$(b_4)^{+1}$, 457.241$(b_5)^{+1}$, 212.103$(b_3\text{-}H_20)^{+1}$, 555.325$(a_7\text{-}H_20)^{+1}$, 394.238$(y_8)^{+2}$, 350.722$(y_7)^{+2}$, 86.097 (Imm of Leu), 60.045 (Imm of Ser)
	TQLPSAYTNSFTR (P5-Spike)	743.3707	5.0	1256.627$(y_{11})^{+1}$, 1143.543$(y_{10})^{+1}$, 959.459$(y_8)^{+1}$, 725.358$(y_8)^{+1}$, 175.119$(y_1)^{+1}$, 628.818$(y_{11})^{+2}$, 572.276$(y_{10})^{+2}$, 230.114$(b_2)^{+1}$, 343.198$(b_3)^{+1}$, 136.076 (Imm of Tyr), 129.114(Imm of Arg)
